# SyRI: finding genomic rearrangements and local sequence differences from whole-genome assemblies

**DOI:** 10.1186/s13059-019-1911-0

**Published:** 2019-12-16

**Authors:** Manish Goel, Hequan Sun, Wen-Biao Jiao, Korbinian Schneeberger

**Affiliations:** 1 Max Planck Institute for Plant Breeding Research, 50829 Cologne, Germany; 2 Faculty of Biology, LMU Munich, 82152 Planegg-Martinsried, Germany

**Keywords:** Genome comparison, Structural rearrangements, Structural variations, Variant calling, Genome alignments, Genetics, Genome assembly

## Abstract

Genomic differences range from single nucleotide differences to complex structural variations. Current methods typically annotate sequence differences ranging from SNPs to large indels accurately but do not unravel the full complexity of structural rearrangements, including inversions, translocations, and duplications, where highly similar sequence changes in location, orientation, or copy number. Here, we present SyRI, a pairwise whole-genome comparison tool for chromosome-level assemblies. SyRI starts by finding rearranged regions and then searches for differences in the sequences, which are distinguished for residing in syntenic or rearranged regions. This distinction is important as rearranged regions are inherited differently compared to syntenic regions.

## Background

Genomic differences form the basis for phenotypic variation and allow us to decipher evolutionary past and gene function. Differences in genomes can range from single nucleotide differences to highly complex genomic rearrangements, and they are commonly described as local sequence differences in comparison to a reference sequence. But even though the annotation of all sequence differences against a reference sequence would be sufficient to reconstruct the actual sequence of a genome, sequence differences alone cannot describe the complex genomic rearrangements. For example, a translocation is a genomic rearrangement where a specific sequence has moved from one region in the genome to another region. Although such a translocation could be described as a deletion at one region and an insertion at the other region, this annotation would miss the information that the deleted/inserted sequence is the same and that the deleted sequence is actually not deleted but rather relocated to a different region. Like translocations, inversions and duplications also introduce differences in the genome structure by changing location, orientation, and/or copy number of specific sequences. But even though this information is usually not considered when analyzing whole-genome sequencing data, differences in genome structure are relevant as they can be the basis for diseases phenotypes [1], reproductive strategies [2–4], and survival strategies [5].

Many of the state-of-the-art methods used to predict genomic differences utilize short or long read alignments against reference sequences [6]. Even though such alignments allow to find local sequence differences (like SNPs, indels, and structural variations) with high accuracy, accurate prediction of structural differences remains challenging. In contrast, whole-genome assemblies enable the identification of complex rearrangements as the assembled contigs are typically much longer and of higher quality as compared to raw sequence reads [7]. However, despite recent technological improvements to simplify the generation of whole-genome de novo assemblies [8], there are so far only a few tools which use whole-genome assemblies as the basis for the identification of genomic differences [9]. Available tools include AsmVar, which compares individual contigs of an assembly against a reference sequence and analyzes alignment breakpoints to identify inversions and translocations [10]; Assemblytics, which utilizes uniquely aligned regions within contig alignments to a reference sequence to identify various types of genomic differences including large indels or differences in local repeats [11]; and Smartie-sv, which compares individual alignments between assembly and reference sequences [12].

Here, we introduce SyRI (Synteny and Rearrangement Identifier), a method to identify structural as well as sequence differences between two whole-genome assemblies. SyRI expects whole-genome alignments (WGA) as input and starts by searching for differences in the structures of the genomes. Afterwards, SyRI identifies local sequence differences within both the rearranged and the non-rearranged (syntenic) regions. SyRI annotates the coordinates of rearranged regions (i.e., breakpoints on both sides of a rearrangement in both genomes) providing a complete regional annotation of rearrangements. This is a significant improvement compared to current methods which typically do not predict both breakpoints for all rearrangements in both of the genomes [13–15].

Moreover, commonly used tools have limited functionality in identifying transpositions (i.e., the relocation of a sequence within a chromosome) and distal duplications. SyRI provides an efficient method for accurate identification of all common rearrangements including transpositions and duplications. For simplicity, unless specified otherwise, we refer to transpositions and translocations together as “translocations” and “duplications” refer to both distal and tandem duplications.

Finally, we validate SyRI’s performance with simulations and in comparison with existing tools developed for the identification of genomic differences. We also apply SyRI to divergent genomes of five model species, including two *Arabidopsis thaliana* strains, for which we experimentally validate over 100 predicted translocations.

## Results

### The hierarchy in genomic differences

Genomes can differ in structure as well as in sequence. *Differences in structure* occur if highly similar regions have different copy numbers, locations, or orientations between different genomes. Here, we will refer to these regions as rearranged regions, whereas all conserved regions are referred to as syntenic. In contrast, *differences in sequence* are variations in the nucleotide sequence resulting in SNPs, indels, and so on.

**Fig. 1:**
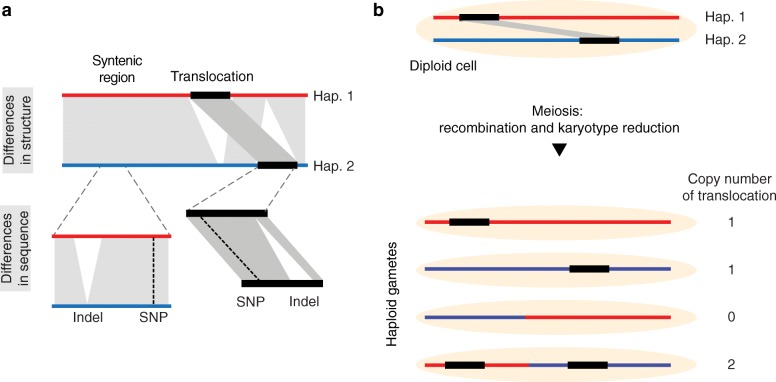
Hierarchy of genomic differences and their propagation. **a** Genomic differences include differences in the structure (like inversions, translocations, or duplications) as well as local sequence differences like SNPs/indels. Differences in sequence can occur in syntenic regions as well as in rearranged regions. **b** A diploid cell containing two haplotypes, which can be distinguished by a translocation. Following meiosis and recombination, the haploid gametes can feature different copy-number variations for the translocated regions and the sequence differences in it

It is important to note that differences in sequence can occur in both, syntenic as well as rearranged regions (Fig. 1a). This introduces a hierarchy into the variations in genomes where, for example, a SNP can be present within a translocated region. Even though resequencing analyses usually do not distinguish between sequence differences in syntenic versus rearranged regions, this distinction is important as some rearranged regions (and the local sequence differences in them) do not follow Mendelian segregation patterns in the offspring. Instead, due to the different locations in a genome, the inheritance of rearrangements can lead to changes in copy number or even loss of the rearranged regions (Fig. 1b).

### How SyRI works

**Fig. 2:**
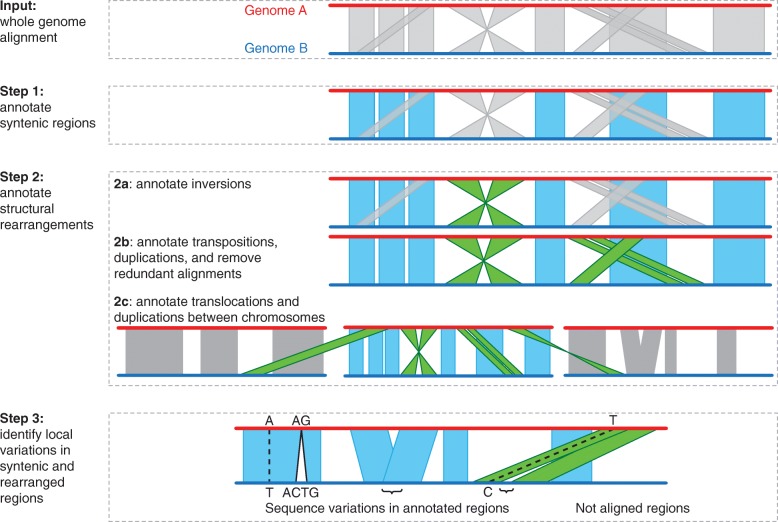
Workflow for the identification of genomic differences. SyRI uses whole-genome alignments (WGA) as input. A WGA consists of a set of local alignments, where each local alignment (gray polygon) connects a specific region in one genome to a specific region in the other genome. Step 1: SyRI identifies the highest scoring syntenic path between the corresponding genomes (blue alignments). The syntenic path represents the longest set of non-rearranged regions between two genomes. Step 2 (a–c): The remaining alignments are separated into structural rearrangements and redundant alignments. Structural rearrangements (green alignments) are classified into inversions, transpositions, and duplications, and finally inter-chromosomal rearrangements. Step 3: Local differences in the sequences are identified in all syntenic and rearranged regions. SNPs and small indels are parsed directly from the local alignments, whereas more complex sequence variations (e.g., like large indels and CNVs) are identified in the overlaps and gaps between consecutive local alignments. Also, all non-aligned regions in between syntenic and rearranged regions are reported for completeness

SyRI is a whole-genome comparison tool that annotates differences in structure and sequence between two whole-genome assemblies (Fig. 2). It starts by identifying all syntenic regions between the two genomes. Since all non-syntenic regions are rearranged by definition, identifying syntenic regions identifies rearranged regions at the same time (Fig. 2: Step 1). In a second step, SyRI groups the rearranged regions into inversions, translocations, and duplications (Fig. 2: Step 2). As the last step, SyRI identifies sequence differences within both rearranged and syntenic regions (Fig. 2: Step 3).

To perform these three steps, SyRI generates different *genome graphs* from the local alignments from a pairwise whole-genome alignment (WGA). Here, we used the MUMmer3 toolbox to perform WGA [16, 17], but other alignment tools like minimap2 [18] can be used as well (Additional file 1: Note 1). In the following, we describe the individual steps of SyRI in more detail.

#### Step 1: Syntenic region identification

SyRI identifies syntenic regions by selecting the longest, non-contradicting subset of aligned regions which are all syntenic to each other. For this, it selects all forward alignments between a pair of homologous chromosomes and generates a genome graph in the form of a directed acyclic graph (DAG) (Additional file 1: Note 2, Figure S1). SyRI then uses dynamic programming to identify the highest scoring path from the nodes that represent one end of a chromosome to the nodes that represent the other end (using similar algorithms as implemented in MUMmer [19, 20]). This process is repeated for each pair of homologous chromosomes.

#### Step 2a: Inversion identification

An inversion is defined as a set of inverted alignments in between two syntenic alignments (Additional file 1: Figure S2). Reverse complementing the alignments of one of the genomes makes inversions align similarly to syntenic alignments. Following this idea, SyRI selects all inverted alignments between a pair of corresponding chromosomes and reverse complements one of the chromosomes (Additional file 1: Figure S3, Note 3). Then, analogous to the syntenic path identification, SyRI again builds up a genome graph using these new forward alignments. From this graph, SyRI infers all possible candidate inversions between the two genomes (Additional file 1: Figure S3a). However, as candidate inversions can overlap and result in conflicting annotations (Additional file 1: Figure S3b), SyRI compares the annotations of all candidate inversions simultaneously and selects the best set of non-overlapping non-conflicting inversions while maximizing the overall alignment score of the two genomes.

#### Step 2b: Translocation and duplication (TD) identification

After synteny and inversion identification, all remaining alignments are either footprints of TDs or are redundant (repetitive) alignments (Additional file 1: Note 4, Figure S4-S7). SyRI analyzes these alignments to find TDs while removing redundant alignments. For this, SyRI first groups the alignments such that each group represents all alignments of a putatively rearranged region (candidate TD) (Additional file 1: Figure S5, S6). Each candidate TD is given a score based on its alignment length and gap length between consecutive alignments. Low scoring candidates and those that are overlapping with syntenic or inverted regions are filtered out.

As a result of repeats, rearranged regions can have different candidate TDs aligning to different copies of the same repeat region. Therefore, overlapping candidate TDs often result in conflicting annotations. SyRI resolves these overlapping candidate TDs by selecting the non-conflicting subset of candidate TDs with the highest alignment score (Additional file 1: Note 4, Figure S5, S7).

##### Grouping of alignments to generate annotation blocks

After identifying syntenic and rearranged alignments, SyRI combines all neighboring alignments of the same type to form annotation blocks. For example, a syntenic block would contain all consecutive syntenic alignments. Likewise, inversion or TD blocks include all alignments which together form the extent of an inversion or a TD.

#### Step 3: Identification of sequence differences

SyRI annotates small variations (like SNPs and small indels) which are found in the local alignments generated by the whole-genome alignment algorithm as well as larger structural variations (like indels or CNVs), which are not part of the local alignments. To find these structural variations, SyRI analyzes the gaps and overlaps between all consecutive alignments in annotation blocks and identifies indels, highly divergent regions (HDRs), and CNVs/tandem repeats (Additional file 1: Figure S8) similar to the SV identification of Assemblytics [11]. Finally, SyRI also reports all *un-aligned regions* which are not part of any annotation block.

### Performance evaluation using simulated genomes

We simulated 600 rearranged genomes by randomly inserting inversions, transpositions, translocations, tandem duplications, distal duplications, and indels into the reference genome of *A. thaliana* (the “Methods” section). We used these genomes to compare SyRI’s performance with six other tools. These included tools based on whole-genome assemblies like AsmVar, smartie-sv, and assemblytics as well as tools which required long reads (sniffles and picky) or short reads (LUMPY) as input [10–15]. For the tools that required sequencing reads data as input, we simulated reads from the simulated genome and aligned them to the reference sequence (the “Methods” section). For all assembly-based methods, we used the simulated genomes directly. Since each of the tools annotated rearrangements in a slightly different manner, we introduced different categories of success to unify their performance similar to an earlier study [13]: a structural rearrangement was considered to be “identified” when all breakpoints were identified together (as one annotation) and had correct annotation, “indicated” when at least one breakpoint was identified with correct annotation, “incorrect” when at least one breakpoint was identified but the annotation was wrong, and “missed” when none of the breakpoints was identified (Additional file 1: Figure S9). For indels, we compared the location and size of the predicted and simulated variations. As the assembly-based methods were not designed to identify all different types of rearrangements, we assessed their performance only for rearrangements which they were designed for.

**Fig. 3:**
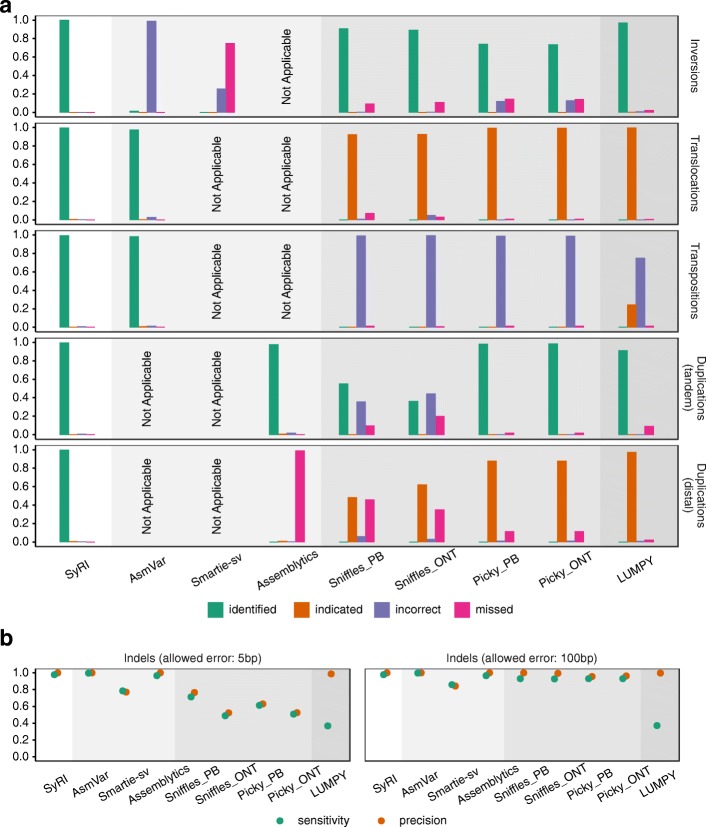
SyRI’s performance compared to six other tools on simulated assemblies. **a** Ratio of rearrangements accurately predicted by each of the tools. **b** Sensitivity (green points) and precision (orange points) values for the prediction of indels. For indels, we compared the location and size of the predicted indels with the simulated indels, allowing for error in both location and size. Two different error limits were used: 5 and 100 bp. Values are averages from the analysis of 100 simulated genomes. “Not Applicable” implies that the specific tool is not designed to identify the specific genomic difference. Background colors represent the data type required by the respective tools (from white to dark gray: chromosome-level de novo assembly, de novo assembly, long sequencing reads (both PacBio (PB) and Oxford Nanopore (ONT) reads), short sequencing reads)

In our analysis, SyRI identified most of the rearrangements accurately (Fig. 3a). AsmVar performed well for identification of transpositions and translocations, but both AsmVar and Smartie-sv were not able to identify inversions correctly. Assemblytics was able to find most of the tandem duplications correctly, but missed distal duplications. All read-based methods showed similar performance. These methods could identify many of the simulated inversions and tandem duplications; however, for rearrangements involving relocation of genomic regions in the two genomes (transpositions, translocations, and distal duplications), these tools were not able to identify rearrangements correctly. For translocations and distal duplications, these tools indicated the presence of these variations; however, they either could not identify all breakpoints or could not identify them as one rearrangement. For transpositions, these methods could find breakpoints; however, the breakpoints were typically not annotated as transpositions. False-positive rates were low in general (Additional file 1: Figure S10) except when identifying transpositions and distal duplications. All tools identified indels with high sensitivity and precision; however, assembly-based methods (SyRI, AsmVar, and Assemblytics) were generally more accurate (Fig. 3b).

This analysis showed that SyRI can predict rearrangements with high accuracy. It can identify all possible rearrangements and provide complete information about the corresponding breakpoints in both genomes. This advantage of SyRI results from the different identification strategy of SyRI, which is based on full-length assemblies that are not required by other tools.

### Performance evaluation using real genomes

To test SyRI’s performance with real data, we applied it to identify the variations in the human genome NA19240 for which gold standard variation data were recently published (Additional file 1: Figure S11, the “Methods” section) [21]. These gold standard variation data include differences which were predicted based on whole-genome shotgun read alignments against the reference sequence and, therefore, include variations from both haplotypes of this genome. In addition, a whole-genome assembly, which includes only one of the two haplotypes, is available as well [22]. Using this whole-genome assembly in comparison to the reference sequence, SyRI identified 55.2% (9685 out of 17,545) of the gold standard insertions, 54.5% (9494 out of 17,391) of the deletions, and 49.7% (81 out of 163) of the inversions (Additional file 1: Figure S12, the “Methods” section), which is consistent with the presence of only one of the haplotypes in the assembly. In comparison to the other tools tested here, SyRI identified a higher proportion of different types of genomic variations of the gold standard variation data (Additional file 1: Figure S12).

For a second comparison, we generated a chromosome-level assembly of the (homozygous) *A. thaliana* L*er* genome using long PacBio reads. The assembly CN50 and CL50 values (chromosome number normalized N50 and L50 values) were 12.6 Mb and 1 respectively (Additional file 2: Table S1, the “Methods” section, Additional file 1: Figure S13) [23]. We again applied the other tools to identify differences between the Col-0 and L*er* genomes (Additional file 1: Figure S14, the “Methods” section). For read-based methods, we observed falsely annotated deletions and tandem duplications (Additional file 2: Table S2), which were in fact transpositions and distal duplications, but were mis-annotated as large local variations (Additional file 1: Figure S15).

### Effect of genome contiguity

SyRI requires whole-genome alignments from chromosome-level assemblies as input. If one or both of the assemblies is/are incomplete, pseudo-chromosomes can be generated using homology between the assemblies themselves or using homology to a chromosome-level reference sequence using tools like RaGOO (Additional file 1: Note 5, [24]). To analyze the effect of the contiguity of the original assembly on SyRI’s performance, we performed a simulation analysis where we first generated multiple incomplete assemblies from the chromosome-level assembly of *A. thaliana* L*er* by randomly breaking the chromosome-level scaffolds in unconnected pieces (the “Methods” section). These scattered assemblies were then reassembled with RaGOO using their homology to the *A. thaliana* Col-0 reference genome.

We then identified rearranged regions in each of these re-assemblies by comparing them to the reference sequence using SyRI. This was then compared to the results SyRI generated when comparing the original chromosome-level assembly of L*er* against the reference sequence.

**Fig. 4:**
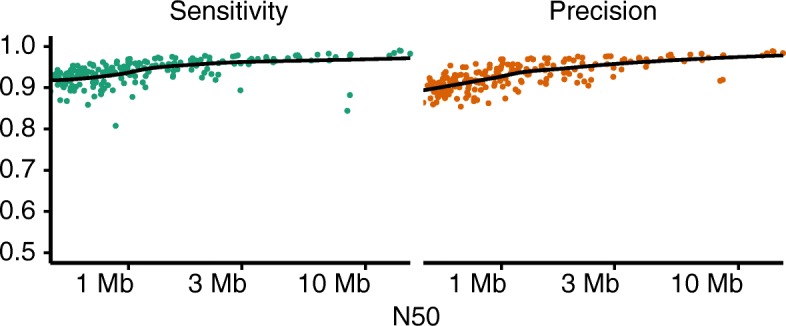
Rearrangement identification from incomplete assemblies. SyRI’s performance for the identification of rearranged regions from incomplete assemblies. Points represent simulated incomplete assemblies, and the black lines represent the polynomial fit

More than 90% of the assemblies with N50 of more than 470 kb (before the homology-based reassembly) had a sensitivity of more than 0.9 (Fig. 4). Similarly, more than 90% of the assemblies with N50 more than 674 kb had a precision of more than 0.9. The shortest assemblies we generated had N50 values in the range of 470–500 kb, and the predictions based on these assemblies still had average sensitivity and precision values of 0.92 and 0.90 respectively.

We then evaluated SyRI’s efficiency in identifying rearranged regions when both genomes are at scaffold level. For this, we generated scattered assemblies from both the Col-0 reference sequence and the L*er* assembly. Since current pseudo-chromosome generation tools only concatenate scaffolds of one assembly using homology with another assembly, we developed a heuristic script to generate homology-based pseudo-chromosomes using two incomplete assemblies (Additional file 1: Note 5). As before, we identified rearranged regions from these pseudo-genomes and compared them to the rearranged regions identified between the full-length assemblies. For assemblies with N50 values of more than 868 kb and 721 kb, sensitivity and precision values were more than 0.7 in more than 70% of the cases (Additional file 1: Figure S16). For assemblies with lower contiguity (N50: 470–500 kb), the average sensitivity and precision were 0.56 and 0.65, respectively.

Together, this shows that the prediction of genomic rearrangements is nearly complete even if one of the genomes is not on chromosome-level, but has assembly contiguity of N50 > 500 kb. If both assemblies are not on chromosome-level, the quality of the predictions is reduced; however, it is still possible to get useful insights on a subset of the rearrangements.

### Runtime estimation when comparing human, yeast, fruit fly, and maize genomes

To analyze SyRI’s runtime performance, we searched for intra-species genomic differences in four different model organisms: human, yeast, fruit fly, and maize (Additional file 2: Table S1). For its application to human genomes, we compared whole-genome assemblies of NA12878 and NA19240 against the reference genome GRCh38.p12 [22, 25]. For yeast, we compared the de novo assembly of strain YJM1447 against the reference genome from strain S288C [26, 27]. For fruit fly (*Drosophila melanogaster*), the de novo assembly of strain A4 was compared to the reference genome [28, 29]. For maize, we compared the de novo assembly of PH207 against the B73 reference genome [30, 31]. To limit computational requirements, we masked the highly repetitive maize genome while all other genomes were analyzed without masking [32].

In each comparison, including human, at least 5% of the assembled genomes were found to be non-syntenic (Table 1, Additional file 1: Figure S17–S21). The CPU runtime for the smaller and simpler yeast genomes was 34.5 s, whereas for the two human genomes SyRI took ~ 10 min, while memory usage was less than 1 GB for each of the comparisons (Table 1) (without considering SNPs and small indels parsing). The exception was the comparison of the repetitive maize genomes, which took ~ 1 h of CPU time and ~ 6GB of RAM. Since SyRI considers all alignment combinations, the runtime and memory usage can be high in repetitive genomes (Additional file 1: Note 6 and Figure S22). However, the number of alignments can be drastically reduced by decreasing the WGA sensitivity (i.e., omitting small, 10–100 s bp alignments), which in turn decreases runtime and memory consumption of SyRI.

**Table 1:** Structural differences identified by SyRI and corresponding computational resources

Species	Sample	Assembly size	CPU runtime (in seconds)	Memory usage (in MB)		Syntenic regions	Structural rearrangements			Un-aligned
							Inversion	Translocation	Duplication	
Human	NA12878	3.03 Gb	542.71	581	Size	2.8 Gb	7.0 Mb	11.6 Mb	27.9 Mb	224.1 Mb
					% genome	91.1	0.2	0.4	0.9	7.4
					Number	1147	66	270	3766	840
	NA19240	3.04 Gb	528.79	1003	Size	2.8 Gb	3.7 Mb	11.8 Mb	27.1 Mb	208.8 Mb
					% genome	91.7	0.1	0.4	0.9	6.9
					Number	1134	68	254	3429	848
Yeast	YJM1447	12.1 Mb	34.51	5	Size	11.2 Mb	1.8 kb	92.0 kb	629.6 kb	87.3 kb
					% genome	92.5	0.02	0.8	6.0	0.7
					Number	222	3	54	370	164
Fruit Fly	A4	135.5 Mb	522.02	289	Size	124.8 Mb	119.5 kb	2.0 Mb	7.5 Mb	1.2 Mb
					% genome	92.1	0.1	1.4	5.5	0.8
					Number	1947	15	636	4387	1365
Maize	PH207	2.06 Gb	3342.62	5873	Size	1.3 Gb	82.5 Mb	10.1 Mb	15.9 Mb	669.6 Mb
					% genome	62.2	4.0	0.5	0.8	32.5
					Number	8779	195	3954	9612	15,166

### Experimental validation

**Fig. 5:**
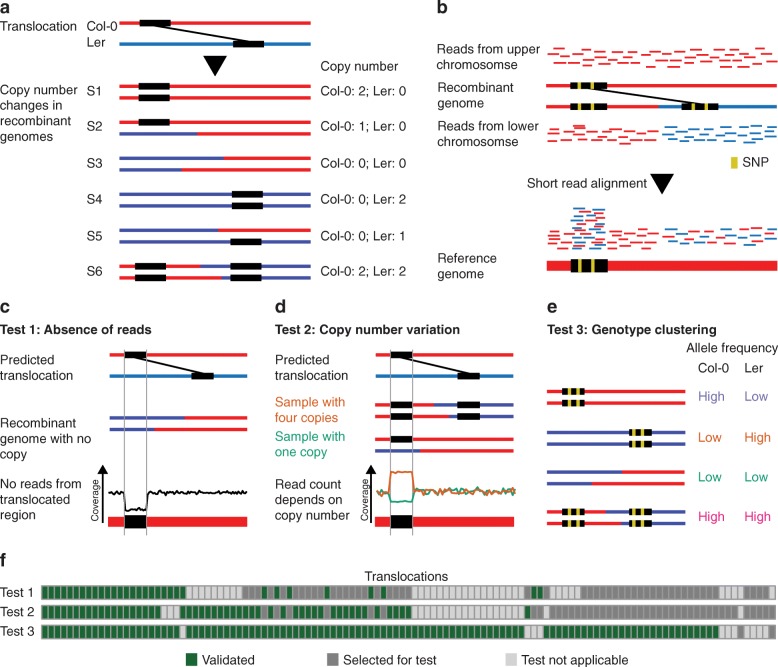
Recombination introduces copy-number variation. **a** Recombination between two haplotypes with translocated regions can lead to copy-number differences in the recombined genomes. **b** Such differences can be observed by aligning short-read sequencing data from recombinant genomes to the reference genome. **c**–**e** Three different tests to assess the existence of the predicted translocations have been applied. These included **c** testing for the absence of reads in samples with no copy of the translocated DNA, **d** goodness-of-fit between expected copy number and observed copy number, and **e** clustering of samples with the same genotypes at the translocation. **f** In the heatmap, columns correspond to individual translocations and rows correspond to the three different tests, while the color of a cell represents whether a translocation was validated (green), was selected but could not be validated (dark gray), or was filtered out as the test was not applicable (gray)

To validate some of the predicted translocations in the genome of *A. thaliana* L*er*, we used a genetic approach which was based on the observation that recombinant offspring genomes feature different copy numbers of translocated DNA (Fig. 1b; 5a), while non-translocated regions always occur with the same copy number. The actual copy number of translocated DNA in a recombinant genome relies on the genotypes at the two insertion sites of the translocation. For example, translocated DNA is duplicated if the two insertion sites of a translocation are combined into one recombinant haplotype.

We used available whole-genome sequencing data of a set of 50 F_2_ recombinant plants, which were generated by crossing Col-0 and L*er*, followed by self-pollination of the resulting F_1_ hybrids [33]. We aligned the short reads (~ 5x genome coverage/sample) to the Col-0 reference sequence and used the genotypes at ~ 500 k SNP markers to reconstruct the parental haplotypes using TIGER (Fig. 5b) [34, 35].

Based on this haplotype information, we estimated the expected copy number for 117 translocations, which were larger than 1 kb, in each of the samples. The expected copy number was then compared to the outcome of three different tests. The first two tests were based on the assumption that all reads from a translocated region align to the same loci in the reference genome independent of the actual location of the rearranged region in the sequenced sample (Fig. 5b) [36]. This allows estimating copy number of a translocation using read coverage in the respective region of the reference. For the first test, we analyzed the absence of reads in translocated regions in recombinant genomes, which were predicted to feature no copy of the translocated region (Fig. 5c) (using 0.2x read coverage as a cut-off to distinguish between absence or presence of a translocation). For the second test, we assessed the goodness-of-fit between expected copy number and observed copy number for a translocation across all recombinants (as estimated from the normalized read counts in the translocation regions; Fig. 5d; the “Methods” section). The third test was based on the sequence differences between the different alleles of a translocation. For this, we tested differences in the read counts supporting either the Col-0 (or L*er*) alleles of a translocation. Depending on the copy number of the different alleles of a translocation, the allele count should also vary. In consequence, samples with the same genotypes at the two loci of a translocation should have similar allele counts, whereas samples with different genotypes should also show different allele counts (Fig. 5e; the “Methods” section).

Out of 117 translocations, 108 (92.3%) could be confirmed by at least one test (Fig. 5f). We manually checked the read alignments of the nine translocations that could not be confirmed and found support for the existence of each of the translocations, which however had not been strong enough to be identified by any of the three test criteria. In summary, this supports that a large majority of the translocations predicted by SyRI are real.

## Discussion

We introduced SyRI, a tool that identifies genomic differences between two whole-genome assemblies. The genomic differences include structural differences as well as differences in sequences. However, instead of identifying differences directly, SyRI starts by identifying all syntenic regions between the genomes, as all other (non-syntenic) regions are rearranged by definition.

Once the structural rearrangements are found, SyRI identifies local sequence differences in both syntenic and rearranged regions. The identification of local sequence differences in rearranged regions introduces a hierarchy of genomic variations (e.g., SNPs in translocated regions). This distinction is important as rearranged regions are differently inherited as compared to syntenic regions. If this is not accounted for, genotypes in rearranged SNPs can confound the interpretation of genomic patterns during selection screens, genome-wide association, or recombination analysis [37, 38]. SyRI now offers a straight-forward solution to filter SNPs in rearranged regions assuming whole-genome assemblies are available.

Compared to sequencing reads, whole-genome assemblies are much more powerful in predicting genomic rearrangements. SyRI utilizes the information in whole-genome assemblies to identify all breakpoints in both reference and query genomes, providing a more comprehensive and accurate annotations compared to read alignment-based methods. Finally, though SyRI is based on a genome graph that is built up from the local alignments of a WGA, this algorithm can be easily adapted for rearrangement identification in other types of genome graphs as well [39, 40].

## Conclusions

We have developed SyRI which, to our knowledge, is the first tool to identify all structural and sequence differences between two chromosome-level genome assemblies. Its novel approach is highly efficient and provides a classification of sequence differences for being in syntenic or rearranged regions. Using SyRI, we identified genomic rearrangements and sequence differences in humans, *A. thaliana*, fruit fly, yeast, and maize genomes. Additionally, we validated the existence of more than 100 predicted translocations. SyRI is available as an open source tool and is being actively developed and improved.

## Methods

### Long read sequencing of the genome of *A. thaliana* L*er*

*A. thaliana* L*er* plants were grown in the greenhouse at the Max Planck Institute for Plant Breeding Research. DNA was extracted using the NucleoSpin® Plant II Maxi Kit from Macherey-Nagel. We used the PacBio template prep kit > 20 kb for Sequel systems (SMRTbell Template Prep Kit 1.0-SPv3) with damage repair (SMRTbell Damage Repair Kit -SPv3) and BluePippin size selection for fragments > 9/10 kb. Sequencing of two SMRT cells was done with the Sequel Sequencing Plate 1.2 and the Sequel Binding Kit 1.0. Movie Time 360 min.

### Assembly generation

We filtered the PacBio reads (removed size < 50 bp or QV < 80 reads) using SMRTLink5 and generated de novo assembly using Falcon, Canu, and MECAT [41–43]. We polished the assemblies using Arrow from SMRTLink5, used SAMTools to identify small assembly errors, and then removed them with Illumina short reads mapping using BWA [44, 45]. We selected the Falcon-based assembly as it showed the highest assembly contiguity. Using whole-genome alignment between Falcon and Canu or MECAT assemblies, we further joined few contigs. Contigs aligning to multiple chromosomes were split if the conflicting region was not supported by Illumina short reads. The contigs from organellar DNA sequences were removed, and all others were anchored into pseudo-chromosome based on homology with the reference sequence. Adjacent contigs were connected with a stretch of 500 “N” characters. To note, the assembly of the L*er* accession was also described in a recent study (preprint [46]).

### Whole-genome alignments

All assemblies used in this work were filtered to select only chromosome-representing scaffolds (unplaced scaffolds were removed). We used the *nucmer* alignment tool from the MUMmer toolbox [17] to perform WGAs. Nucmer was run with --maxmatch to get all alignments between two genomes and also included -c, -b, and -l parameters which were selected to balance alignment resolution and runtime based on genome size and number of repeat regions (full commands are available in Additional file 2: Table S3). Alignments were filtered using the *delta-filter* tool, and the filtered delta files were converted to the tab-delimited files using the *show-coords* command. Before whole-genome alignments, both maize genomes were masked using RepeatMasker v4.0.6 [47].

### Simulating rearranged genomes

We simulated structural rearrangements in the *A. thaliana* reference genome using the *R* package *RSVSim* and SURVIVOR [48, 49]. We simulated 40, 436, 100, 100, and 1241 events for inversions, transpositions, translocations, tandem duplications, and distal duplications respectively, and for each rearrangement, 100 genomes were simulated. For inversions, transpositions, and distal duplications, the number of rearrangements and their corresponding sizes were sampled from real differences found between the Col-0 and L*er* genomes. For tandem duplications, the size of the duplicated region ranged from 100 to 1000 bp, whereas translocations ranged from 1000 to 5000 bp long. For simulating indels, we used SURVIVOR to simulate 100 genomes containing 1000 indels in the range of 1–500 bps.

From these rearranged genomes, we simulated PacBio and Nanopore reads using SURVIVOR. We used the *A. thaliana* long read data generated by Michael et al. (NCBI project accession: PRJEB21270) to generate read profiles required by SURVIVOR and simulated reads to get a 30x coverage [50]. Short reads were simulated using wgsim (parameters used: -e 0.001 -d 550 -N 12000000 -1 150 -2 150) to get 30x coverage [51]. All reads were aligned to the A. *thaliana* reference genome using minimap2, and the alignments were converted from SAM to BAM format and sorted using samtools [18, 44].

### Running tools on simulated genomes

SyRI: Genome assemblies were aligned using nucmer (Additional file 2: Table S3), and SyRI was run with default parameters. Assemblytics: We used the same alignments generated by nucmer as used for SyRI. The default value for unique sequence length was used, and variants size was set from 1 to 100,000 bp. AsmVar: The tool was run based on the demo script provided with the tool. For genome alignment, lastdb was run using the default parameters, whereas lastal and last-split were run using the parameters provided in the demo [52]. Similarly, variants were detected using the ASV_VariantDetector tool of AsmVar with the default parameters. Smartie-sv: The pipeline was run using the default settings. However, the number of jobs to be run in parallel and job wait time was adjusted to make it suitable for the computer resources available. Sniffles: Sniffles was run separately for PacBio and Nanopore simulated reads using the default parameters. Alignments were generated through minimap2 and converted to BAM and sorted using samtools. Picky: Picky was run using the same methodology and parameters as described by the authors for both PacBio and Nanopore reads. LUMPY: Reads were aligned by minimap2, and the alignments were pre-processed using samblaster [53] and samtools as per the instructions provided by the authors. While running LUMPY, paired-end read distribution parameters were changed to match the simulated reads (mean 550, read_length 150, min_non_overlap 150).

Breakpoints predicted by tools were considered to match the simulated rearrangement if they were within ± 150 bps range. For simulated translocations and transpositions, reads-based method did not predict any translocation; however, they predicted breakends which matched the predicted translocations, therefore, we considered these breakends as representative for translocations. For duplications (distal and tandem), all annotation types resembling duplications were considered. For indels, we compared the location and size of the predicted indels with the simulated indels, allowing for error in both location and size. Two different error limits were checked: 5 and 100 bp.

### Performance evaluation with real genome data

For both the *A. thaliana* (L*er*) and the human (NA19240) genome, we used the same methods as above to simulate sequencing reads from whole-genome assemblies, to perform alignments with the reference genomes, and to identify genomic differences. For human genomes, we used the error profiles provided by SURVIVOR [49]. Count and sizes of the variations were extracted from the output files using in-house scripts. For the AsmVar comparison of Col-0 vs L*er*, we used the .svd output file instead of the .vcf output file as the former had better annotations. An indel was considered as identified if there was a simulated indel of the same type (insertion or deletion) within 100 bp of the location of the predicted indel and the size difference between two indels was not more than 100 bps.

### Comparison with the gold standard variation dataset

Variant calls for the gold standard dataset were downloaded from the NCBI [21]. The variants were generated with an older version human reference genome (GRCh38) and were therefore re-mapped to the newer GRCh38.p12 version of the human reference genome using the NCBI Genome Remapping Service. An indel from the gold standard dataset was considered to be identified if a predicted indel of the corresponding type existed within the surrounding 100 bp. For inversion predictions, we checked the overlap between inversions from the gold dataset and the inversions, inverted translocations, and inverted duplications as annotated by SyRI.

### Pseudo-chromosome generation and output comparison

We generated 200 fragmented assemblies of the L*er* genome by introducing 10–400 random breakpoints. Pseudo-genomes were generated for each of the fragmented assemblies using RaGOO with default parameters. Additionally, we generated 100 fragmented assemblies each of Col-0 and L*er* again by introducing 10–400 random breakpoints. These fragmented assemblies were assembled by a heuristic script (Additional file 1: Note 5) to generate pseudo-molecules. For 16 assemblies, pseudo-molecule generation failed and these samples were skipped from further analysis. A genomic rearrangement identified from the pseudo-genomes was considered to be correct if the same rearrangement type was present within 100 bp up or downstream.

### Data extraction and transformation of the 50 recombinant genomes

For validation, we used whole-genome sequencing data of 50 F_2_ recombinant plants that we generated recently [33]. We extracted allele count information from consensus call files generated by SHORE [54]. For each predicted translocation, we estimated its copy number as the ratio between average read coverage for the translocated region and the average read coverage across the entire genome of the respective sample. Translocations in the centromeric regions and for which more than 25% of the translocated sequence had at least 10% reads with Ns were filtered out. For allele count analysis, we selected high-confidence (25 bp conserved in both directions) SNPs in translocated regions as markers.

### Validation of translocations: absence of reads (test 1)

We selected F2 samples which, according to predicted genotypes, should have lost the translocated DNA and thus should not give rise to any reads from the translocated region. Only translocations for which at least two samples that had lost the translocated regions existed were tested. And only those translocations for which all tested samples had no reads were considered as validated.

### Validation of translocations: expected vs. observed copy number (test 2)

For each translocation, we selected samples which had different genotypes at the two associated loci for the translocation. This removes some of the samples with two copies and helps to remove a bias towards genomes with a copy number of two, which can affect this test. We further selected translocations for which we found samples with at least three different copy-number values predicted. A linear model was fit using the *lm* function in *R*. *p* values for the model-fit were adjusted for multiple testing using the *BH* method [55], and translocations for which adjusted *p* values were less than 10^−6^ and slope more than 0.75 were considered as valid.

### Validation of translocations: genotype clustering (test 3)

Allele count values at the SNP markers were normalized and outliers (markers having very high allele counts) were removed. Translocations were tested only when they had at least two different classes of samples (genotypes) with each class having at least three samples and at least three SNP markers in the translocated regions. Translocations for which alternate allele counts did not change across the samples (variance < 1) were also filtered out.

#### Cluster fit calculation

First, the distance between two samples was defined as the Euclidean distance between their reference allele counts and alternate allele counts. Then, the *closeness_score* was calculated as the sum of ratios of the average distance between the samples belonging to a genotype to the average distance to samples of other genotypes.

#### Simulating distributions

Background distributions for the *closeness_score* were simulated by generating random clusters. For each sample, allele counts (reference and alternate) were sampled using a Poisson distribution. For true translocations, the *closeness_score* would be low as samples from the same genotype would be much closer to each other, whereas samples from different genotypes would be far. For each translocation, we calculated the lower-tail *p* value of retrieving the corresponding *closeness_score*. *p* values were adjusted for multiple testing using *BH* method, and translocations with *p* value < 0.05 were considered valid.

## Supplementary information

**Additional file 1.** Additional notes and figures - Additional notes describing the method and additional results

**Additional file 2.** Additional tables - Information about methodology and data used, and additional results

**Additional file 3.** Review history

## Data Availability

The assembly of the L*er* genome has been submitted to the European Nucleotide Archive (http://www.ebi.ac.uk) and is publicly available under the accession number GCA_900660825 [56]. The reads are available as part of a separate study under the project ID PRJEB31147 (preprint [46]). All other assemblies are publicly available at NCBI (https://www.ncbi.nlm.nih.gov/), and their accession numbers are GCA_000001735.3 [57], GCA_000001405.27 [25], GCA_002077035.3 [22], GCA_001524155.4 [22], GCA_000146045.2 [27], GCA_000977955.2 [26], GCA_000001215.4 [29], GCA_002300595.1 [28], GCA_000005005.6 [31], and GCA_002237485.1 [30]. Further details about the assemblies are in Additional file 2: Table S1. BAM files for the 50 F_2_ recombinant genomes are available at European Nucleotide Archive under the project ID PRJEB29265 [33]. SyRI is freely available under the MIT license and is available online [58]. The version of SyRI used in this work is available at doi.org/10.5281/zenodo.3555197 [59]. SyRI is developed using Python3.5 on Linux and can run on other operating systems as well.
