# Supplementary material for: SyRI: finding genomic rearrangements and local sequence differences from whole-genome assemblies
Source: Genome Biol. 2019 Dec 16;20:277. doi: 10.1186/s13059-019-1911-0 (PMC6913012; doi:10.1186/s13059-019-1911-0)
Supplement: Supplementary file 3 — Additional file 3. Review history [file 13059_2019_1911_MOESM3_ESM.docx]

**1^st^ round**

**Reviewer 1**

With this manuscript the authors present SyRI, an efficient tool for genome-wide identification of structural rearrangements (SR) from genome graphs, which are built up from pair-wise whole-genome alignments. Instead of searching for differences, SyRI starts by finding all co-linear regions between the genomes. As all remaining regions are SRs by definition, they can be classified as inversions, translocations, or duplications based on their positions in convoluted networks of repetitive alignments.

Using graph based genomes for variant discovery is an important problem. However the manuscript and the method are not appropriately described. What about deletions - are those discovered as well ? The authors do compare to AsmVar and Assmblytics but not to other popular SV calling software, or use publicly available and annotated genomes such as those from the GIAB consortium. NA19240 has been shown to have a much larger number of predicted variations and should be used as a good test for the performance of the method (see Chaisson et al, Nature Communications 2019) What about experimental validation of the predictions ?

Please consider the following questions:

-Is the Software likely to be of broad utility? Is it easy to install and use?

--- Software has potential to be of broad utility.

-Does the software represent a significant advance over previously published tools (as demonstrated by direct comparison with available related software)?

--- Has not been established

-Please indicate briefly the novel features and/or advantages of the software and/or please reference the relevant publications and which alternatives, if any, it should be compared with.

--- Need to be compared with other software tools such as Picky/Sniffles etc

-Are the methods appropriate to the aims of the study, are they well described, and are necessary controls included?

--- Methods need to be better described. Figure 1 and Figure 2 are not referenced/described well enough in the text

-Are the conclusions adequately supported by the data shown?

--- No

-Are sufficient details provided to allow replication and comparison with related analyses that may have been performed?

--- Yes

-Is the paper of broad interest to others in the field, or of outstanding interest to a broad audience of biologists?

--- Paper is potentially of wide interest however the performance has not been demonstrated.

**Reviewer 2**

The authors present a method for detection SVs between genome assemblies using a graph. The paper is well written, the results are compelling, and there is a need for this type of method as de novo genome assemblies become more common.

I was able to download and install the software:

Notes:

- g++ is required, but not listed as a dependency.

- after python setup.py install, syri/bin/syri is not executable, I had to chmod +x syri/bin/syri

I was not able to test the software. There are a number of steps that must be complete leading up to the execution of syri and they are not well described. A step-by-step example of how to recreate the analysis in the manuscript would be helpful. All steps should be include, from downloading the data to getting the results given.

My main complaint about this method is that it does not detect deletions/insertions, which are major sources of genetic diversity. Since the nodes of the graph are based on pair-wise alignments, sequences that only appear in one genome are not even represented in the graph. Strangely, in the comparison with Assemblytics and AsmVar, the authors seem to claim that SyRI can find deletions/insertions. Also, Figure S4 includes insertions and deletions. Clarity here would be greatly appreciated.

The comparison here for SVs only considers AsmVar. Kronenberg, Science 2018 did genome-to-genome SV detection on Apes and the method they provide is being used by others in the field. It would seem appropriate to compare this method to something more recent and from a more high-profile project. Also, AsmVar was only considered in the simulated data. The results of SyRI on Eol-O and Ler need to be compared to results from AsmVar, and ideally newer methods

I agree that short-read SV callers have gaps, but there are many examples of how long-read callers can do quite a bit better. Since the genomes considered here are from long reads, I would like to see a comparison to methods such the one from Sedlazeck Nature Methods 2018.

I am confused by the description of the algorithm that creates the DAG. Nodes are connected if the two underlying alignments are co-linear and no other co-linear alignment is between them. From this, it would seem that the nodes must be co-linear in both genomes. But in Fig3a, nodes "a" and "e" are connected but are not co-linear in either genomes. Nodes "a" and "c" are connected, but are co-linear in just Genome A. In the same figure, "d" and "e" are not connected, but they are co-linear in Genome A. Similarly, nodes "g" and "i" are not connected. There is some discussion in the figure legend, but there is not enough information in there for me to understand. I am also not sure about how inverted alignments affect edges between nodes. In Fig 3bi there is an edge between "b" and "c", where "c" is an inverted alignment, but in the same figure "a" and "b" are not connected. More detail on the graph construction is needed.

I found the TD identification difficult to follow, and while the additional materials did provide great levels of detail, I found them mostly confusing. Please consider including a figure that walks through this process.

**Response to reviewers**

**Reviewer #1**

With this manuscript the authors present SyRI, an efficient tool for genome-wide identification of structural rearrangements (SR) from genome graphs, which are built up from pair-wise whole-genome alignments. Instead of searching for differences, SyRI starts by finding all co-linear regions between the genomes. As all remaining regions are SRs by definition, they can be classified as inversions, translocations, or duplications based on their positions in convoluted networks of repetitive alignments.

Using graph based genomes for variant discovery is an important problem. However, the manuscript and the method are not appropriately described.

# We thank the reviewer for acknowledging the importance of using graph-based genomes for variant discovery. In order to describe the method better, we have now introduced multiple supplementary notes describing the individual steps of the algorithm in more detail, while two new sections of the main text (“The hierarchy in genomic differences” and “How SyRI works”) explain the idea of (the individual steps of) the method.

## What about deletions - are those discovered as well ?

# Yes, SyRI also finds deletions and insertions.

*SyRI identifies genomic differences on two different levels. First, SyRI finds broad* ***differences in the genomic structure*** *including inverted, translocated and duplicated regions. Once this is done, SyRI finds all* ***differences in sequence****, including SNPs and small indels as well as large indels, highly divergent regions, CNVs etc (Response-Figure 1).*

***Response-Figure 1.*** *SyRI finds two layers of genomic differences. The first layer includes differences in the structure (upper panel), while the second layer includes differences in the sequence between the two genomes (lower panel). Differences in sequence can occur in rearranged as well as in non-rearranged regions.*

*As* ***differences in sequence*** *are found across the entire genome, they can be distinguished for residing in non-rearranged (i.e. syntenic) or rearranged regions (for example a SNP can be in a syntenic region or in a translocation). Though this difference is usually not considered, it is an important difference, as rearranged regions are differently inherited as compared to syntenic regions and have been implicated in various phenotypes.*

## The authors do compare to AsmVar and Assmblytics but not to other popular SV calling software, or use publicly available and annotated genomes such as those from the GIAB consortium. NA19240 has been shown to have a much larger number of predicted variations and should be used as a good test for the performance of the method (see Chaisson et al, Nature Communications 2019)

# We thank the reviewer for this valuable suggestion. In addition to AsmVar and Assemblytics, we now compare SyRI also with another whole-genome assembly based tool (Smartie-SV) as well as tools that are based on read alignments including Sniffles, Picky, and LUMPY (Figure 3, Additional File 1: Figure S9, S10, S12). The individual comparisons were limited to the type of genomic differences that are predicted by the respective tools and by SyRI, as none of the other tools predicted exactly the same types of genomic differences as SyRI. Moreover, none of the tools addresses the different layers of genomic differences (Figure 1).

# As suggested, the performance comparisons now include a comparison of SyRI and the other tools on the gold standard variations for the human genome NA19240 provided by Chaisson et al. (Additional File 1: Figure S12). This showed that SyRI is able to identify a higher proportion of all different types of genomic variation of the gold standard variation compared to other tools (Additional Figure S10).

## What about experimental validation of the predictions?

# In the manuscript, we describe the experimental validation of ~100 translocations found in the comparison of two divergent Arabidopsis genomes. This was not performed with PCR amplification of the translocated sequence in conjunction with the surrounding region as this would only prove that the translocation is residing in a divergent region, but not that the translocation is indeed in a different region of the genome (which is the fundamental characteristic of a translocation).

# Instead, we used a genetic test to prove that the translocated sequence resides in two different regions of the genomes. The test was based on the observation that recombinant genomes feature different copy numbers of translocated DNA (Response-Figure 2), while non-translocated regions do not change in copy-number.

***Response-Figure 2:*** *Schematic of the inheritance of a translocated region. On top two haplotypes (Col-0 and Ler) can be distinguished by a translocation (black region). In recombinant offspring (including recombined chromosomes of the two haplotypes) we can observe different copy-numbers of the translocated region. As a matter of course, syntenic (non-rearranged) regions do not change in copy-number in offspring genomes.*

*We analysed the genomes of 50 recombinant genomes of the divergent accessions and used three different tests to check whether the regions which were annotated as translocations by SyRI are indeed showing different copy numbers. This validated most (93%) of the translocations, while the remaining translocations showed the expected patterns as well, when we checked them manually.*

-Is the Software likely to be of broad utility? Is it easy to install and use? 
--- Software has potential to be of broad utility.

## -Does the software represent a significant advance over previously published tools (as demonstrated by direct comparison with available related software)? --- Has not been established

## -Please indicate briefly the novel features and/or advantages of the software and/or please reference the relevant publications and which alternatives, if any, it should be compared with. --- Need to be compared with other software tools such as Picky/Sniffles etc

*SyRI is (to our knowledge) the first tool that thoroughly combines the identification of differences in the genomic structures and differences in the sequences and thereby establishes a first framework for the hierarchy of genomic differences (Figure 1a).*

# We now show performance comparisons of SyRI with six other tools illustrating that SyRI is the only tool that can identify distal translocations and duplications and that SyRI out-performs most of the other tools for inversion and indel identification as well.

## -Are the methods appropriate to the aims of the study, are they well described, and are necessary controls included? --- Methods need to be better described. Figure 1 and Figure 2 are not referenced/described well enough in the text

# We have updated the manuscript to better explain the method. This includes changes in the description of the algorithmic details (Additional File 1: Note 2-4, and Figures S1, S3 and S7) and changes in the description of the main concepts in several sections of the main text. We agree with the assessment that the Figure 1 and 2 were not very well referenced in the text. We have added sections “The hierarchy in genomic differences” and “How SyRI works” to better describe the figures in the text.

## -Are the conclusions adequately supported by the data shown? --- No

*We hope that with the new analysis and comparison of SyRI’s performance we can convince the reviewer that SyRI introduces a significant advance to the field of genome comparison.*

## -Are sufficient details provided to allow replication and comparison with related analyses that may have been performed? --- Yes

## -Is the paper of broad interest to others in the field, or of outstanding interest to a broad audience of biologists? --- Paper is potentially of wide interest however the performance has not been demonstrated.

# We would like to thank the reviewer for sharing our enthusiasm and for the helpful comments in general.

# We have added further descriptions on the comparison of SyRI with six other tools using real as well as simulated genomes. We also assessed SyRI’s performance in comparison to the gold standard variation data for the human genome NA19240. We hope that these new analyses help to demonstrate SyRI’s performance.

## **Reviewer #2** The authors present a method for detection SVs between genome assemblies using a graph. The paper is well written, the results are compelling, and there is a need for this type of method as de novo genome assemblies become more common. I was able to download and install the software:  Notes:  - g++ is required, but not listed as a dependency.

## - after python setup.py install, syri/bin/syri is not executable, I had to chmod +x syri/bin/syri

# We thank the reviewer for mentioning these issues. We have updated the installation guide and hope that SyRI can now be successfully installed (<https://schneebergerlab>.github.io/syri/install.html).

## I was not able to test the software. There are a number of steps that must be complete leading up to the execution of syri and they are not well described. A step-by-step example of how to recreate the analysis in the manuscript would be helpful. All steps should be include, from downloading the data to getting the results given.

# We appreciate that the reviewer tried running SyRI to assess its performance. We acknowledge the difficulty faced by the reviewer and therefore we have added a step by step example on SyRI’s github website (<https://schneebergerlab.github.io/syri/pipeline.html>) as well as on the github repository (<https://github.com/schneebergerlab/syri/blob/master/example/pipeline.sh>). We hope that this will help in running SyRI successfully.

## My main complaint about this method is that it does not detect deletions/insertions, which are major sources of genetic diversity. Since the nodes of the graph are based on pair-wise alignments, sequences that only appear in one genome are not even represented in the graph. Strangely, in the comparison with Assemblytics and AsmVar, the authors seem to claim that SyRI can find deletions/insertions. Also, Figure S4 includes insertions and deletions. Clarity here would be greatly appreciated.

# Indeed SyRI also identifies deletions and insertions.

*SyRI identifies genomic differences on two different levels. First, SyRI finds broad* ***differences in the genomic structure*** *including inverted, translocated and duplicated regions. For this SyRI uses genome graphs which are build up from the local alignments of the whole-genome alignment. Multiple local alignments are combined together (as annotation blocks) such that each of these blocks represents one rearrangement (e.g. the local alignments which together form an inversion).*

*Once this is done, SyRI finds all* ***differences in sequence****, including SNPs and small indels (which are found in the local alignments) as well as large indels, highly divergent regions, CNV etc (which are found in the breaks between the local alignments of each annotation block - similar to the method of Assemblytics).*

*As* ***differences in sequence*** *are found across the entire genome, they can be further distinguished for residing in non-rearranged (i.e. syntenic) or rearranged regions (for example, a SNP can be in a syntenic region or in a translocation). Though this difference is usually not considered, it is an important difference, as rearranged regions are differently inherited as compared to syntenic regions.*

*Please see Response-Figure 1 in the responses to reviewer 1 for a graphical overview of the two layers of genomic differences described by SyRI.*

*(As this point was also raised by reviewer 1 we have introduced major changes to the description of the methods in the main and supplementary texts. We hope that both idea and method are more clear now.)*

The comparison here for SVs only considers AsmVar. Kronenberg, Science 2018 did genome-to-genome SV detection on Apes and the method they provide is being used by others in the field. It would seem appropriate to compare this method to something more recent and from a more high-profile project. Also, AsmVar was only considered in the simulated data. The results of SyRI on Eol-O and Ler need to be compared to results from AsmVar, and ideally newer methods

# We thank the reviewer for the valuable suggestion. In addition to AsmVar and Assemblytics, we now also compare SyRI with Smartie-SV (Kronenberg, Science 2018), Sniffles, Picky, and LUMPY (Figure 3). We compared the performances of all these tools using simulated data and real genomes including a human genome and two Arabidopsis genomes (Additional File 1: Figure S9, S10, S12). These analyses showed that SyRI is able to identify a higher proportion of all different types of genomic variation of the gold standard variation compared to other tools and that other tools are limited by severe overcalling of large genomic differences.

## I agree that short-read SV callers have gaps, but there are many examples of how long-read callers can do quite a bit better. Since the genomes considered here are from long reads, I would like to see a comparison to methods such the one from Sedlazeck Nature Methods 2018.

# As described above, we have added comparison of SyRI with six other tools which identify genomic differences using whole-genome assembly (AsmVar, Assemblytics, Smartie-sv) as well as long-read (Sniffles and Picky), and short-read (LUMPY) data.

## I am confused by the description of the algorithm that creates the DAG. Nodes are connected if the two underlying alignments are co-linear and no other co-linear alignment is between them. From this, it would seem that the nodes must be co-linear in both genomes. But in Fig3a, nodes "a" and "e" are connected but are not co-linear in either genomes. Nodes "a" and "c" are connected, but are co-linear in just Genome A. In the same figure, "d" and "e" are not connected, but they are co-linear in Genome A. Similarly, nodes "g" and "i" are not connected. There is some discussion in the figure legend, but there is not enough information in there for me to understand. I am also not sure about how inverted alignments affect edges between nodes. In Fig 3bi there is an edge between "b" and "c", where "c" is an inverted alignment, but in the same figure "a" and "b" are not connected. More detail on the graph construction is needed.

# The reviewer is right that the “the nodes must be co-linear in both genomes” for them to be connected by an edge. However, by “co-linear” we implied that the corresponding aligned regions have the same relative position in both genomes. For this, the alignments need not be closely-linked. That is, an edge would be added from alignment “x” to alignment “y” if the aligned regions of “y” are downstream to the aligned regions of “x” on both genomes and no other co-linear regions is between them. Consequently, in Additional File 1: Figure S1, there is an edge from “a” to “e” because the aligned regions of “e” are downstream to aligned region of “a” on both genomes whereas “d” and “e” are not connected because the aligned region of “e” is downstream to aligned regions of “d” on genome A and upstream on genome B.

# In order to explain these points more clearly, we have updated the method description and in particular, we try to avoid the term ‘co-linear’ to limit any confusion.

# Regarding inversion identification (former Figure 3b), the first step is to reverse complement the query genome to transform all inverted alignments to directed alignments (Additional File 1: Figure S3). A new DAG is then constructed based on these alignments using a similar process as used for syntenic path identification.

# We realize that these questions result from incomplete descriptions of the methods. In order to better explain the algorithm, we have added new additional figures (Additional File 1: Figure S1, S3, S7) and provide a more detailed explanation for algorithms identifying synteny, inversion, and TD identification respectively (Additional File 1: Note 2-4).

## I found the TD identification difficult to follow, and while the additional materials did provide great levels of detail, I found them mostly confusing. Please consider including a figure that walks through this process.

*Once again, thank you very much for your efforts to help to advance this manuscript. We have modified the main text to illustrate the challenges involved in TD identification and how SyRI solves them. The details of this can be found in a new additional note (Additional File 1: Note 4) that explains the TD identification process in more detail.*

**2^nd^ round**

**Reviewer 2**

The authors did a nice job of making the methods section more clear. While adding a more robust comparison also improved the manuscript, I am concerned that nearly every tool did not find translocations or duplications despite many published accounts of these tools in fact finding these SVs. The authors have some cryptic explanation about how other tools have limited definitions of these SVS, which makes me think that this is a semantic not a technical issue. If this result is real, then it is very surprising and the authors need to dig deeper as to why tools that can find these SVs is not finding these SVs.

**Response to reviewers**

**Reviewer #2**

Reviewer #2:

The authors did a nice job of making the methods section more clear.

*We thank the reviewer for acknowledging the improvements made to the manuscript.*

While adding a more robust comparison also improved the manuscript, I am concerned that nearly every tool did not find translocations or duplications despite many published accounts of these tools in fact finding these SVs. The authors have some cryptic explanation about how other tools have limited definitions of these SVS, which makes me think that this is a semantic not a technical issue. If this result is real, then it is very surprising and the authors need to dig deeper as to why tools that can find these SVs is not finding these SVs.

*The reviewer is correct about semantic differences. In response to this comment, we realized that our simulations only include distal duplications, but no tandem duplications (which can be found by other tools). Similarily, we referred to any kind of sequence relocation as translocation. However, we realized that we should distinguish between transpositions (relocation of sequence in one chromosome) as compared to translocations (relocation of sequence to a different chromosome). Using these new definitions we have now repeated our simulations of rearranged regions to test the performance of various different tools.*

*Moreover, similarily to the tool comparison presented by Sedlazeck et al, Nature Methods, 2018, we now distinguish between different ‘degrees of identification’ of the simulated rearrangements. A structural rearrangement was considered to be ‘identified’ when all breakpoints were identified together and have the correct annotation, ‘indicated’ when at least one breakpoint was identified with correct annotation, ‘incorrect’ when at least one breakpoint was identified but the annotation was wrong, and ‘missed’ when none of the breakpoints were identified (Figure R1).*

*As SyRI is based on chromosome-level assemblies, SyRI can annotate the coordinates of the breakpoints of all rearrangements in both genomes. Other assembly-based methods can also annotate all breakpoints, but they are limited in the types of rearrangements they can identify. Read-based methods do not have information about the structure of the query genome and are, therefore, unable to find the breakpoints of a rearrangement in the query genome. In many cases read-based methods do not even connected the breakpoints of one rearrangement within the reference genomes and thus cannot classify the rearrangements correctly. With SyRI, it is now possible to get all of this information (Figure R2).*

*Overall, our simulations clearly outline the advantages of SyRI, which however rely on the use of more comprehensive input information: chromosome-level assemblies. We have added this and more information on the different performances and input requirements of the different tools to the performance evaluation and the Background section of the manuscript.*

*We would like to thank the reviewer again for his comment as we realize that it made us drastically improve our manuscript.*

*Figure R1: Breakpoints of simulated rearrangements. Grey alignments corresponds to conserved regions, white alignments corresponds to simulated rearrangements. Arrows mark the coordintates that were tested to classify the correctness of the predicted rearrangements. For rearrangements in which a region relocates between two genomes, we required annotation of breakpoints in both genomes for an accurate identification of the rearrangement.*

*Figure R2: SyRI’s performance compared to six other tools on simulated assemblies. (a) Ratio of accurately predicted rearrangements. Values are averages from the analysis of 100 simulated genomes. ‘Not Applicable’ implies that the specific tool is not designed to identify the specific genomic difference. Background colors represent the data type required by the respective tools (from white to dark grey: chromosome-level genome assembly (SyRI), genome assembly (AsmVar, Smartie-sv, Assemblytics), long sequencing reads (Sniffles_PB, Sniffles_ONT, Picky_PB, Picky_ONT), short sequencing reads (LUMPY)).*

**3rd round**

**Reviewer 2**

Thanks for the updates. Looks good.
